# Diagnostic value of a coronal STIR sequence in conjoined lumbar nerve root detection: an MRI accuracy study

**DOI:** 10.1007/s00256-025-04945-y

**Published:** 2025-05-14

**Authors:** Georg Wilhelm Kajdi, Thomas Marth, Jung-Ah Choi, Mazda Farshad, Reto Sutter

**Affiliations:** 1 Department of Radiology, Faculty of Medicine, Balgrist University Hospital, University of Zurich, Forchstrasse 340, 8008 Zurich, Switzerland; 2 Swiss Center for Musculoskeletal Imaging, Balgrist Campus AG, Zurich, Switzerland; 3 Department of Radiology, Faculty of Medicine, Hallym University Dongtan Sacred Heart Hospital, Hallym University, Hwaseong, Republic of Korea; 4 Department of Orthopedics, Faculty of Medicine, Balgrist University Hospital, University of Zurich, Zurich, Switzerland

**Keywords:** Conjoined lumbosacral nerve root, CLNR, MRI, Lumbar spine, Accuracy, Coronal imaging, STIR

## Abstract

**Objectives:**

To assess diagnostic accuracy for conjoined lumbosacral nerve root (CLNR) detection on MRI when adding a coronal STIR sequence to the standard lumbar spine protocol.

**Materials and methods:**

In this retrospective study, two radiologists assessed the presence of CLNR and lumbosacral transitional vertebrae (LSTV), using a standard lumbar MRI protocol and an expanded protocol with an additional coronal STIR sequence. Prior radiologist consensus using the expanded protocol served as a reference standard for diagnosis.

**Results:**

In 751 patients (mean age 61.2 ± 15.7 years, 435 females), CLNR was found in 38 patients (5.1%) in consensus. Without coronal STIR, 13 CLNR patients were correctly identified, CLNR was missed in 25 patients, and 3 patients were falsely detected as having one (sensitivity of 34.2%, specificity of 99.6%, positive predictive value (PPV) of 81.3%, negative predictive value (NPV) of 96.6%, and accuracy of 96.3%). With coronal STIR, 31 CLNR patients were correctly identified, CLNR were missed in 7 patients, and one patient was falsely detected as having one (sensitivity of 81.6%, specificity of 99.9%, PPV of 96.9%, NPV of 99.0%, and accuracy of 98.9%). Inter-reader agreement improved from moderate without coronal STIR (*κ* = 0.592; 95% CI 0.38, 0.80) to almost perfect with coronal STIR (*κ* = 0.915; 95% CI 0.84, 0.99). LSTV had a prevalence of 13.3% among patients without and 26.3% among patients with CLNR (*p* = 0.025).

**Conclusion:**

Coronal STIR greatly improved sensitivity and inter-reader agreement for CLNR detection on MRI while slightly improving the specificity and accuracy. A significant association of CLNR and LSTV was found.

**Supplementary Information:**

The online version contains supplementary material available at 10.1007/s00256-025-04945-y.

## Introduction

Conjoined lumbosacral nerve roots (CLNRs) are the most common anomaly involving the lumbar nerve roots, but are overall still a relatively rare finding with no pathognomic clinical tell-tale sign [1, 2]. Even when present, they are often neglected on lumbar spine MR imaging [3]. A widespread prevalence of CLNR is reported in the literature based on surgical, imaging, and autopsy report series, ranging from 1.3 to 14% [4, 5]. The preoperative diagnosis of a CLNR is of clinical relevance in patients undergoing spine surgery [6]: Without preoperative knowledge, CLNRs can be mistaken for part of a herniated disc by the surgeon [7]. Forceful retraction or incision can lead to iatrogenic nerve injury and failed back surgery [8]. Thus, preoperative CLNR identification can change the surgical approach, with adjustments to patient positioning, to the extent of decompression procedures and with the use of specific nerve root sparing access to the disc [4, 6]. In clinical imaging, lumbar MRI has replaced the accurate but invasive CT myelography method for CLNR diagnosis. Both CT myelography and lumbar MRI show higher CLNR detection rates compared to the standard lumbar CT, which underestimates CLNR prevalence [9, 10]. Coronal STIR on lumbar MRI yields a high resemblance to the coronal image reconstructions of CT myelography and has been recommended when CLNR presence is suspected [2, 11]. Despite advocates for a coronal STIR sequence in lumbar spine MRI [12–14], most institutions do not routinely include it. No large cohort study has evaluated the impact of an additional coronal STIR sequence on sensitivity, specificity, and overall accuracy in CLNR detection, compared to a standard MRI protocol that relies only on axial and sagittal image acquisitions.

This study aimed to assess improvement in sensitivity, specificity, and overall accuracy of MRI-based CLNR detection in 751 patients when adding a coronal STIR sequence to the routine lumbar spine protocol.

## Materials and methods

### Patient selection

This retrospective single-center study was approved by the local institutional review board (Kantonale Ethikkommission Zurich) and conducted according to the principles of the Declaration of Helsinki and national ethical standards. All included patients signed the written informed consent that allows their health-related data to be used for research purposes.

**Fig. 1:**
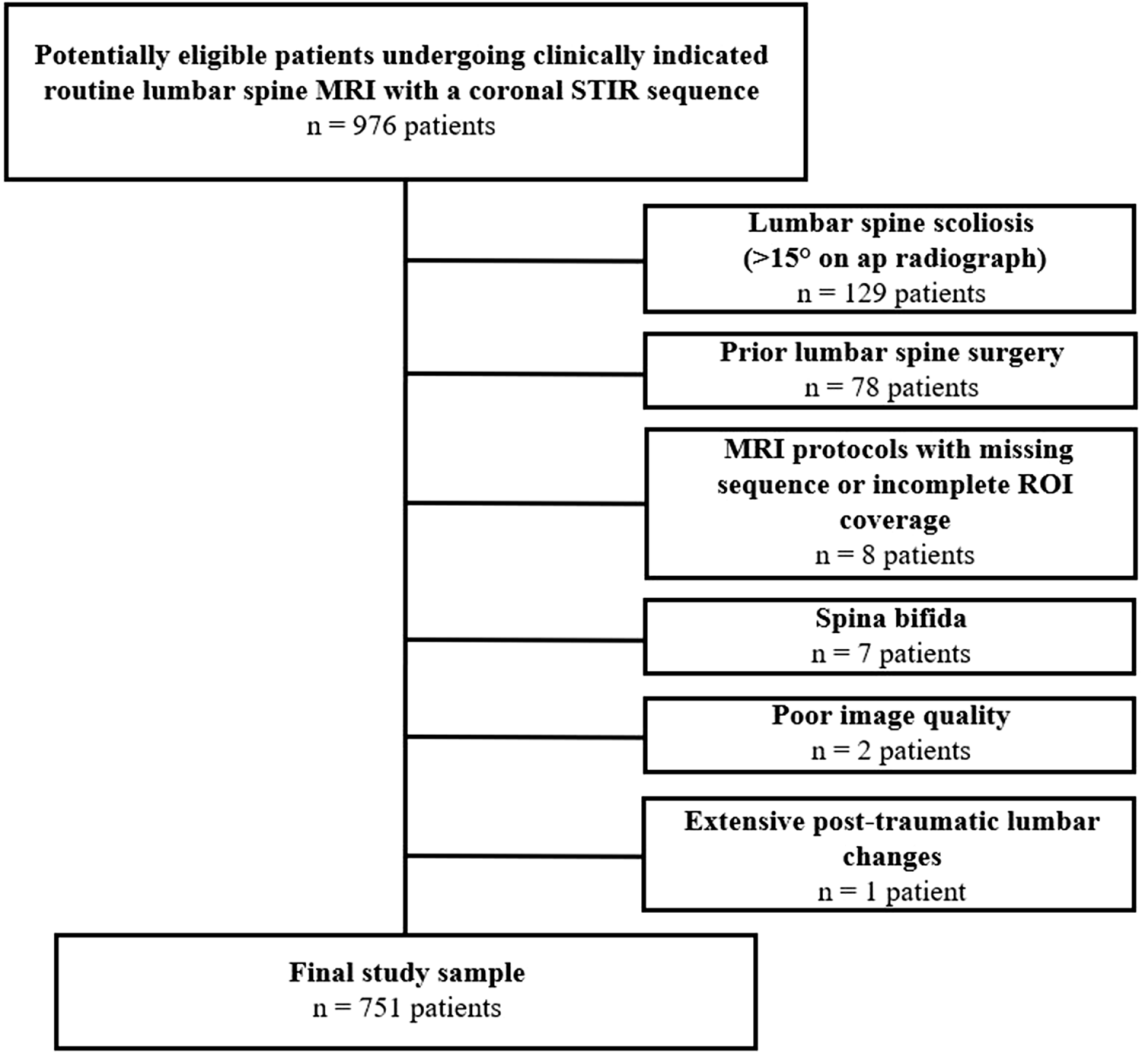
**Flowchart illustrating the patient selection and MRI analysis process.** From n = 976 potentially eligible patients, 225 patients had to be excluded during the selection process, resulting in a final study sample of 751 patients. (ROI, region of interest)

The institutional picture archiving and communication system (PACS) was reviewed for patients > 18 years of age who had undergone lumbar spine MRI within a 3-year time span, using an expanded protocol, including a coronal STIR sequence. Nine hundred seventy-six patients matched the age and imaging inclusion criteria. Two hundred twenty-five patients were excluded from the study. Exclusion criteria entailed lumbar spine scoliosis on radiograph > 15° (*n* = 129), prior lumbar spine surgery (*n* = 78), incomplete MRI protocols or incomplete scans (*n* = 8), spina bifida (*n* = 7), poor image quality (*n* = 2), and extensive post-traumatic lumbar changes (*n* = 1). This resulted in a total of 751 patients included in the study (Fig. 1). Clinical records were reviewed and revealed indications for lumbar MRI to be acute, sub-chronic or chronic lower back pain with and without clinically suspected radiculopathy, neurogenic claudication, sciatica, acute trauma, cauda equina syndrome, and polyneuropathy (Supplementary Table 1).

### Magnetic resonance imaging

Non-contrast MRI of the lumbar spine was conducted on a 3 T unit (SkyraFit, Siemens Healthineers, Erlangen/Germany), using a dedicated 32-channel phased-array spine coil. All patients underwent examination in the supine and head-first positions. The MR protocol consisted of a sagittal T1- and T2-weighted turbo spin echo (TSE), sagittal STIR, axial T2-weighted TSE, and coronal STIR sequence. Detailed scan parameters are summarized in Table 1. The coronal STIR sequence was planned on a sagittal lumbar scout view parallel to the anterior edge of the L3 and L4 vertebral bodies. If their axes were misaligned due to stronger lumbar lordosis, the anterior edge of L3 alone was used for reference. The field of view of the coronal STIR captured the thoracolumbar junction, including the lower two costovertebral joints with the posterior aspect of the lowest two ribs, as well as the lumbosacral transition and sacroiliac joints bilaterally. The lumbar paravertebral space with the iliopsoas muscles and part of the retroperitoneum was included. The protocol, including the tabularized scan parameters (Table 1), was executed according to national and international radiologic society guidelines [15, 16].

**Table 1:** **3 T MRI protocol parameters**

Sequence	T1 TSE Sag	T2 TSE sag	STIR sag	T2 TSE tra	STIR cor
TR (ms)	550	4190	5450	6000	4000
TE (ms)	8.5	92	34	101	37
Bandwidth (Hz/px)	260	270	260	260	260
Slices (*n*)	18	18	18	22	18
Slice thickness (m)	4	4	4	4	4
Spacing (mm)	4.4	4.4	4.4	4.4	4.4
Matrix	384 × 269	448 × 314	384 × 269	384 × 346	320 × 224
FOV (mm)	300 × 300	300 × 300	300 × 300	220 × 220	300 × 300
Pixel spacing (mm)	0.78 × 0.78	0.67 × 0.67	0.78 × 0.78	0.57 × 0.57	0.94 × 0.94
FA	160	139	130	160	130
TA (min:s)	1:10	0:50	1:54	2:12	1:16

*cor*, coronal;* Hz*, hertz;* FA*, flip angle;* FOV*, field of view;* sag*, sagittal;* px*, pixel;* TA*, acquisition time;* TE*, echo time;* TR*, repetition time;* tra*, transversal;* TSE*, turbo-spin-echo

### Image analysis

**Fig. 2:**
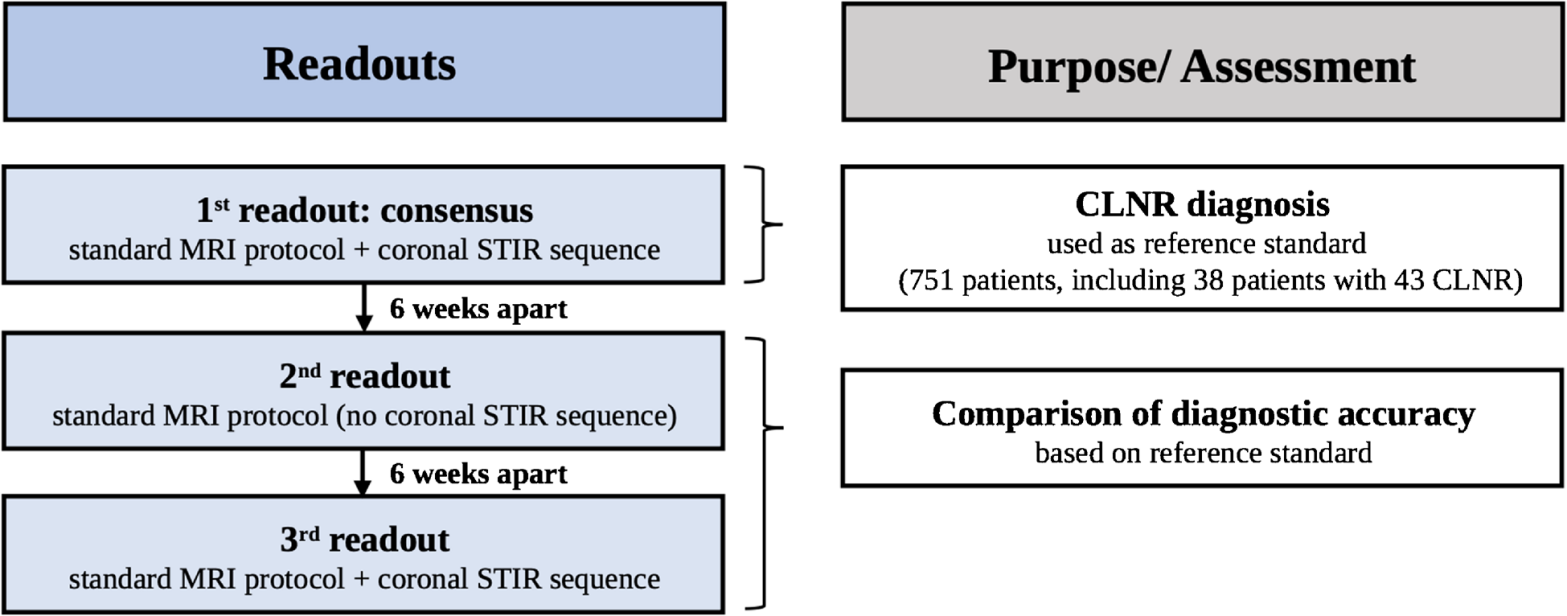
**Flowchart illustrating the readout process.** The first readout by two readers (reader 1 and 2, with 26 and 6 years of experience, respectively) established the consensus of CLNR diagnosis. In ambiguous cases, reader 3 (19 years of experience) was consulted to form a reliable consensus. After the consensus was established, two readers (readers 2 and 4, with 6 and 5 years of experience, respectively) separately carried out readout 2 (without coronal STIR imaging) and readout 3 (including the coronal STIR sequence). To avoid any recall bias, all readouts were set 6 weeks apart from each other. (CLNR, conjoined lumbosacral nerve root; STIR, short tau inversion recovery)

**Fig. 3:**
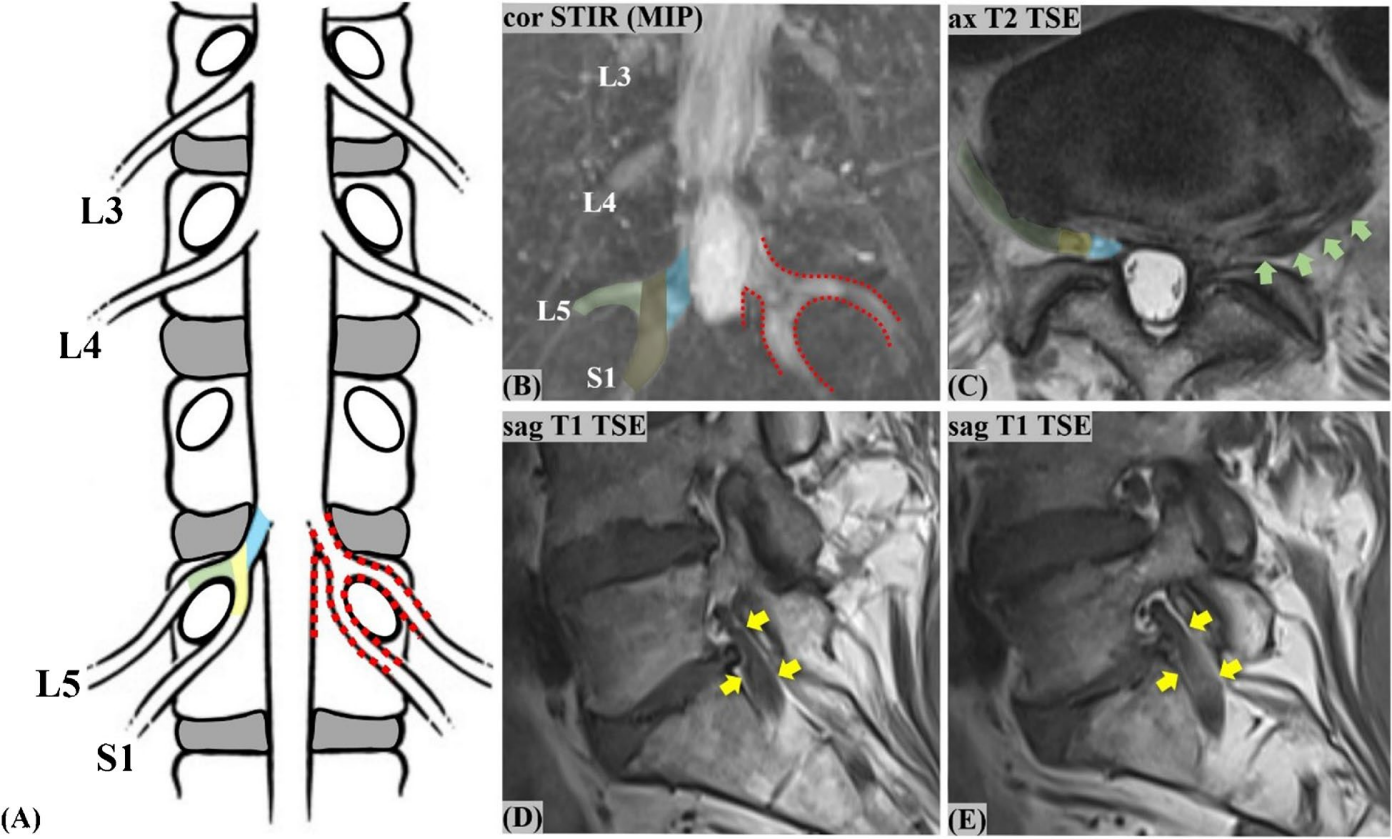
**Conjoined lumbosacral nerve root (CLNR) depiction on MRI, in a rare case of symmetrically affected L5/S1 nerve roots in a 68-year-old female patient.** The schematic (**A**) and coronal STIR MIP (**B**) highlight the bilateral L5/S1 CLNR (both type Ia). The red-dotted line (**A**, **B**) denotes the overall CLNR course, consisting of a common trunk (blue overlay; **A**–**C**), epidural division cranio-medially to the L5-pedicle into the horizontally coursing upper root L5 (green overlay; **A**-**C**) and into the vertically coursing lower root S1 (yellow overlay; **A**–**C**). The horizontal L5 course on the axial T2 TSE (**C**) is the parallel sign (green arrows). The vertical S1 course on the sagittal T1 TSE (yellow arrows; **D**, **E**) in the epidural space behind the disc is the shoulder sign, seen here in two subsequent images. (MIP, maximum intensity projection; STIR, short tau inversion recovery; TSE, turbo spin echo. MIP (**B**) was used for the educational purpose of the illustrations and figures only, and not for CLNR diagnosis)

**Fig. 4:**
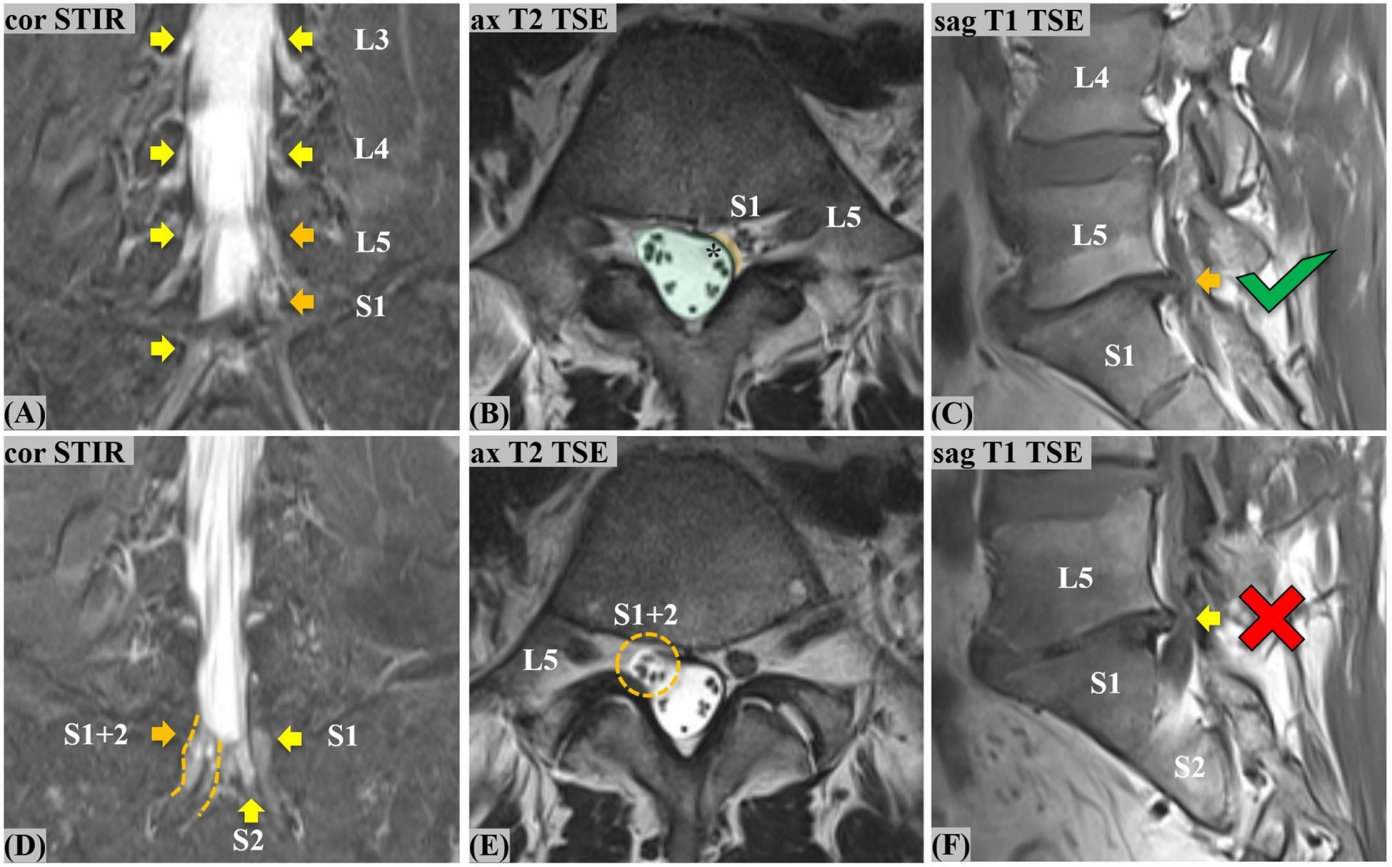
**Asymmetries in conjoined lumbosacral nerve roots (CLNR) and pitfalls in a 51-year old male with multi-segmental CLNR.** Upper row depicts the left L5/S1 CLNR: On coronal STIR (**A**) crowded L5/S1 thecal take-off (orange arrows) on the left contrasts the evenly distributed L3 to S1 roots on the right (yellow arrows), causing asymmetry. Axial T2 TSE (**B**) shows mulitple multiple asymmetries: Prominent epidural fat between thecal sac and epidural S1 root highlights the fat crescent sign (yellow overlay). Ipsilateral blunting antero-laterally, the so-called corner sign, results in a pear-shaped thecal sac (green overlay). Furthermore, there are fewer intra-thecal rootlets on the left (asterisk), due to the early, high-rising left S1 root. Lower row depicts the right S1/S2 CLNR: On coronal STIR (**D**), the thick root sleeve of the right S1 level (orange arrow) shows early epidural re-division (orange dotted line). The axial T2 TSE (**E**) depicts the finding as a collection of supernumerary, over-crowded rootlets within one root sleeve (orange- dotted circle) containing the S1/S2 CLNR. As a potential pitfall, the sagittal T1 TSE (**C**, **F**) demonstrates a true positive shoulder sign on the left (orange arrow) and a false positive shoulder sign on the right (yellow arrow) in the L5/S1 segment

Image analysis was done using a commercially available picture archiving and communication system (PACS) workstation (Merlin, Phoenix-PACS). An initial consensus reading served as the reference standard for the later accuracy assessment. This consensus reading was done using all available sequences of the expanded lumbar spine protocol, including the coronal STIR sequence, and was performed by a fellowship-trained musculoskeletal (MSK) radiologist (G.W.K.) with 6 years of experience and an expert senior MSK radiologist (J-A.C.) with 26 years of experience (Fig. 2). On the axial plane, the imaging diagnosis of CLNR was based on the previously described “parallel sign,” “corner sign,” and “fat crescent sign” [7], as well as other axial asymmetries. Sagittal images were assessed for the “shoulder sign” [7], as well as empty foramina and more than one nerve root in the intervertebral foramen [8]. The “parallel sign” is the horizontal course of a CLNR entirely depicted on a single axial image, parallel to the disc plane [7] (Fig. 3). The “corner sign” describes blunting of the anterolateral corner of the thecal sac adjacent to the exiting CLNR [7] (Fig. 4). The “fat crescent sign” is characterized by crescent-shaped epidural fat, interposed between the thecal sac and the exiting CLNR [7] (Fig. 4). The “sagittal shoulder sign” [7] signifies a vertical structure connecting two consecutive nerve roots and represents a CLNR posteriorly traversing a herniated disc in the epidural space [7] (Figs. 3 and 4).

**Fig. 5:**
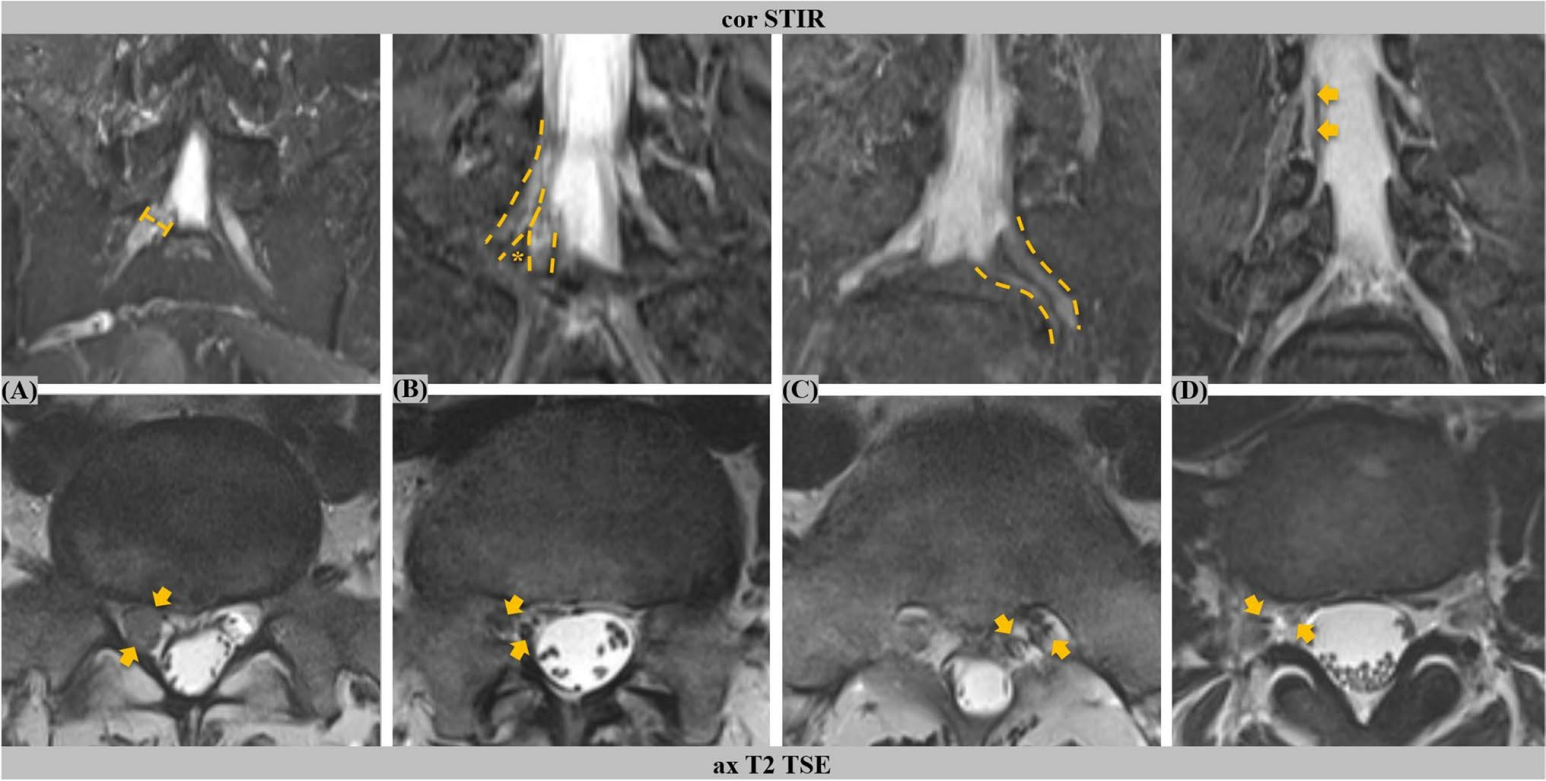
**Case series of 4 different patients, highlighting different findings on coronal STIR imaging of conjoined lumbar nerve roots.** Aside from different heights of thecal take-off of CLNR, other asymmetries such as a wider root sleeve (**A**, CLNR type Ia, orange caliper around L5/S1 on the right), epidural formation of a secondary axilla (**B**, CLNR type Ib, orange asterisk between L4/5 on the right), two rootlets in the same foramen (**C**, CLNR type IIb, orange-dotted lines around L5 on the left) and vertically coursing anastomosing rootlets in the epidural space parallel to the thecal sac (**D**, CLNR type III, orange arrows along L3/L4 anastomosis) can be observable on coronal STIR. Arrows in the lower row (**A**–**D**) indicate the axial T2 TSE correlate of the respective CLNR on the coronal images in the upper row. (CLNR, conjoined lumbosacral nerve root)

**Fig. 6:**
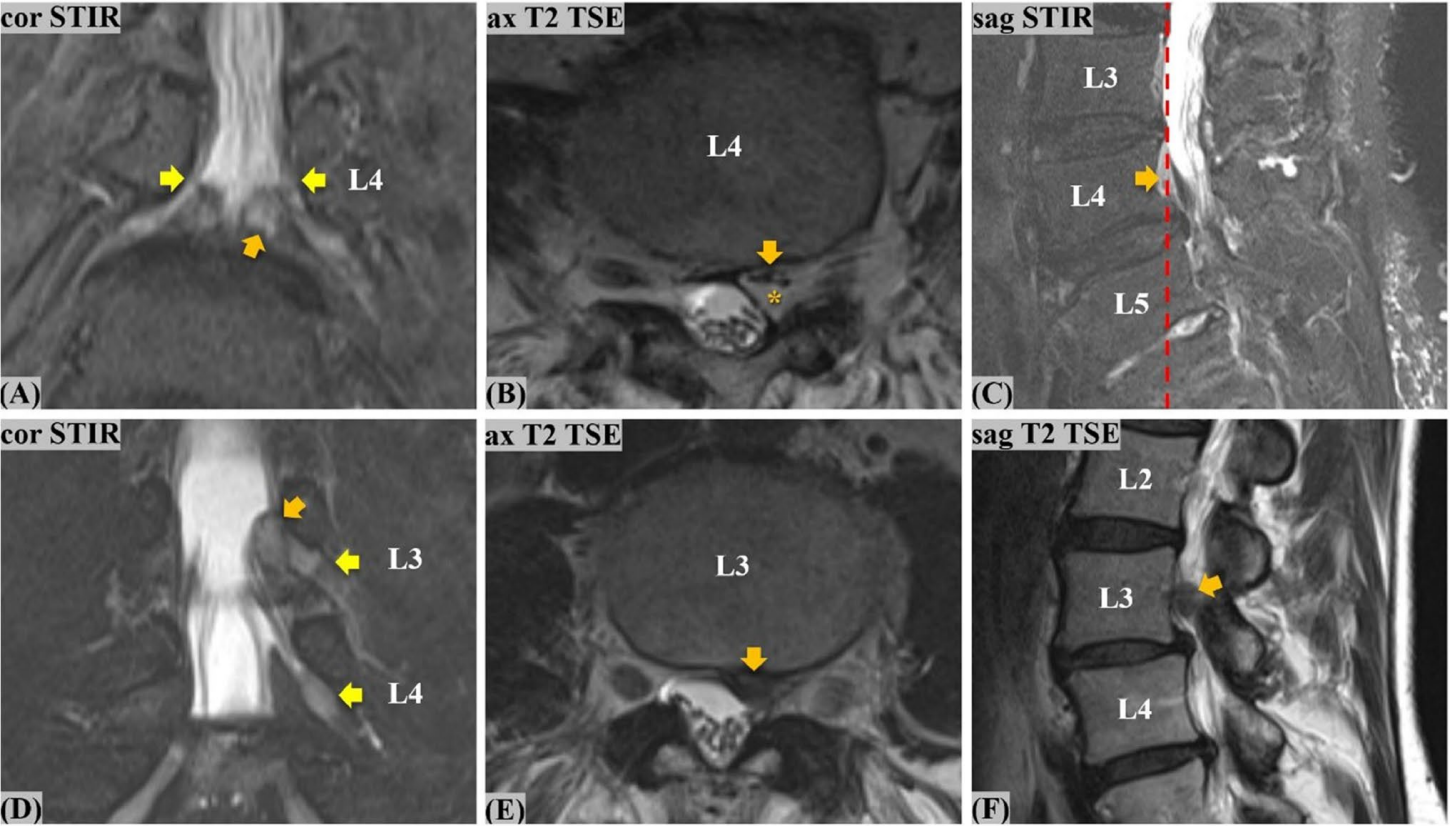
**Two patient examples (upper row: 74-year-old female; lower row: 64-year-old female), illustrating potential pitfalls in coronal conjoined lumbosacral nerve root (CLNR) detection.** Upper row: On the coronal STIR (**A**), L4 nerve roots (yellow arrows) show symmetric thecal take-off, with an alleged CLNR at the left L4 level (orange arrow). Correlating the axial T2 TSE (**B**), the structure proves to be the venous plexus (orange arrow) within asymmetric epidural fat (orange asterisk). Sagittal STIR (**C**) highlights the coronal plane (red-dotted line) running through the venous plexus/epidural fat (orange arrow). Lower row: An elliptic structure (orange arrow) with comparable signal intensity to the L3 and L4 roots on the coronal STIR (**D**) is causing asymmetry. On the axial T2 TSE (**E**), a medio-lateral disc protrusion (orange arrow) is observed. The sagittal T2 TSE (F) proves the structure to be a sequestered disc component (orange arrow). (STIR, short tau inversion recovery; TSE, turbo spin echo)

With reference to the myelography case series [5], the coronal STIR sequence was assessed for asymmetries in height, width, course, or visible extra-thecal division of the exiting nerve roots and of their dural sleeves (Fig. 5). Any of these signs alone or in combination were used for diagnosis. In addition, other etiologies for the aforementioned signs and asymmetries had to be excluded for the CLNR diagnosis still to be made, including herniated discs, a prominent lumbar venous plexus, congenital, or posture-related asymmetries in epidural fat, as well as partial volume effects [17] (Fig. 6). In ambiguous cases, a third reader, another senior MSK expert radiologist (R.S.) with 19 years of experience, was consulted to form reliable consensus. For each scan, it was determined whether CLNRs were present, how many CLNRs were detected, on how many levels and on which side they were found, and which level of nerve roots were involved. Furthermore, we took note of which sequence was subjectively the most helpful in aiding initial CLNR detection for each patient and on which planes the CLNR was detectable overall. Each CLNR was categorized by subtype according to the Neidre and MacNab classification [18] (Supplementary Fig. 1).

**Fig. 7:**
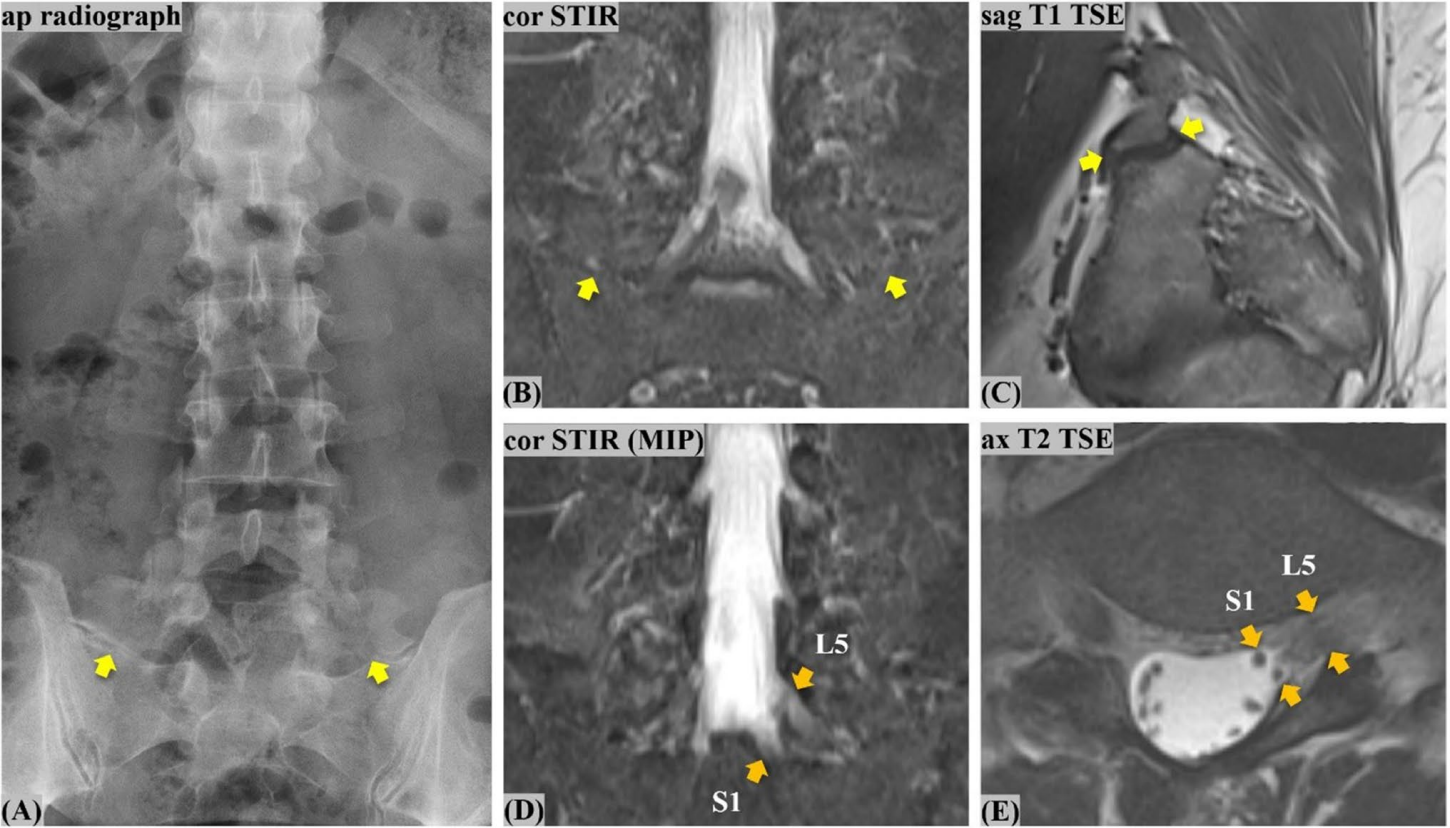
**A 47-year-old, male patient with coinciding lumbosacral transitional vertebra (LSTV) and conjoined lumbosacral nerve root (CLNR).** The LSTV (sacralized L5) is well seen on the ap AP radiograph (**A**), coronal STIR (**B**), and the outer most slice of the sagittal T1 TSE on the right (**C**), showcasing bilateral lumbosacral pseudoarticulation (yellow arrows). Coinciding presence of a CLNR L5/S1 on the left is illustrated on coronal STIR MIP (**D**) and axial T2 weighted TSE (**E**) (orange arrows). (AP, anteroposterior; MIP, maximum intensity projection; STIR, short tau inversion recovery; TSE, turbo spin echo. MIP (**B**) was used for the educational purpose of the illustrations and figures only, and not for CLNR diagnosis)

After the consensus reading, two additional readouts were performed by two fellowship-trained musculoskeletal radiologists (T.M. and G.W.K., with 5 and 6 years of experience, respectively). These readouts were set 6 weeks apart from each other to avoid recall bias (Fig. 2). All MRI studies were anonymized and independently reviewed by the two readers. Examinations were analyzed in random order, whereby both readers were blinded to clinical data. In the second readout, the presence of CLNR was assessed using the standard lumbar spine MRI protocol, without coronal STIR. The third readout was done accordingly, using the expanded protocol including the coronal STIR sequence. Additionally, both readers assessed the presence and subtype of lumbosacral transitional vertebrae (LSTV) according to Castellvi [19] in all patients during the third readout (Fig. 7). LSTV were assessed as follows: with depiction of both the thoracolumbar and lumbosacral transition on the coronal images, each reader diagnosed and classified the LSTV under the assumption of 12 thoracic vertebrae with paired ribs, using the available sequences.

### Statistical analysis

All statistical analyses were performed using SPSS Statistics (v. 29, IBM Corporation, Armonk, New York/USA). Non-normal distribution of data was assessed graphically and analytically using quantile–quantile plots and the Shapiro–Wilk test. Sensitivity, specificity, positive (PPV), negative predictive value (NPV), and overall accuracy assessment for CLNR detection without (2nd readout) and with coronal STIR imaging (3rd readout) relied on the previous consensus reading (1 st readout) as ground truth. In addition to descriptive statistics, the chi-square test was performed to assess differences in gender distribution among patients with and without CLNR and LSTV. McNemar’s test was used to assess differences in CLNR detection between the 2nd and 3rd readout. Differences in age between patients with and without CLNR were assessed with the Mann–Whitney *U* test. Cramér’s V (φc) was calculated for the non-parametric correlation of CLNR and LSTV. Inter-reader agreement was analyzed by kappa statistics (Cohens’ *κ*). The level of agreement was categorized as follows [20]: 0.0 = poor, 0.01–0.20 slight, 0.21–0.40 = fair, 0.41–0.60 = moderate, 0.61–0.80 = substantial, and 0.81–1.00 = almost perfect agreement. All statistical tests were performed two-sided, and a level of significance (*α*) of 0.05 was used.

## Results

### Demographics and patient characteristics

Seven hundred fifty-one patients (a mean age of 61.2. ± 15.7 years, 435 females, Table 2) were included in the study. Thirty-eight out of 751 patients (5.1%) were found to have CLNR in the consensus readout, including 22 women and 16 men, without significant differences regarding gender distribution (*p* = 0.99) of CLNR.

**Table 2:** **Patient and CLNR characteristics**

Patient characteristics	CLNR	No CLNR	*P*-value^1^
Patients (***n*** = 751)	38	713	
Female (***n***, %)	22 (57.9)	435 (57.9)	0.99
Age (years)^2^	61.5 ± 15.6	61.2 ± 15.7	0.36
LSTV (***n***, %)	10 (26.3)	95 (13.3)	0.025
Detected number of CLNR	43	N/A	
CLNR characteristics according to consensus			
Subtypes according to Neidre and McNab (***n***, %)			
Ia	5 (11.6)	N/A	
Ib	21 (48.8)	N/A	
IIa	0 (0)	N/A	
IIb	14 (32.6)	N/A	
III	3 (7.0)	N/A	
Side (***n***, %)			
Unilateral right	16 (42.1)	N/A	
Unilateral left	17 (44.7)	N/A	
Bilateral	5 (13.2)	N/A	
Affected nerve roots/lumbosacral segments (***n***, %)			
L3/L4	6 (13.9)	N/A	
L4/L5	2 (4.7)	N/A	
L5/S1	26 (60.5)	N/A	
S1/S2	9 (20.9)	N/A	
Plane-dependent CLNR detectability (***n***, %)			
Axial detectability (all)	36 (83.7)	N/A	
Solely detectable on the axial	1 (2.3)	N/A	
Sagittal detectability (all)	16 (37.2)	N/A	
Solely detectable on the sagittal	0 (0)	N/A	
Coronal detectability (all)	42 (97.7)	N/A	
Solely detectable on the coronal	4 (9.3)	N/A	
Axial and sagittal detectability combined	13 (30.2)	N/A	
Axial and coronal detectability combined	35 (81.4)	N/A	
Sagittal and coronal combined	16 (37.2)	N/A	
Axial, sagittal, and coronal combined	13 (37.2)	N/A	
Sequence most helpful for initial detection (***n***, %)			
Axial T2 TSE	16 (37.2)	N/A	
Coronal STIR	27 (62.8)	N/A	

*LSTV*, lumbosacral transitional vertebra;* CLNR*, conjoined lumbosacral nerve root;* N/A*, not applicable

^*1*^*Significant results (P* < *.05) are bolded*

^*2*^*Data is given as mean* ± *standard deviation*

### 1st readout: CLNR prevalence and characteristics

In 38 affected patients, a total of 43 CLNR were detected in consensus. Patients showed an even distribution of CLNR among the left and right sides and showed mostly unilateral and unisegmental CLNR presence (33/38; 86.8%). Five of the affected patients (5/38; 13.2%) had two detectable CLNR (Table 2). In 3 of those patients (3/38; 7.9%), the same segment was affected bilaterally (twice L5/S1, once L3/4) (Fig. 3), while in 2 of those patients (2/38; 5.3%), two different segments (each L5/S1 and S1/S2) were affected on opposite sides (Fig. 4). All affected patients (38/38; 100%) had their CLNR distributed along the lumbar and sacral segments L3/4 to S1/S2. Detailed patient and CLNR characterization of the consensus readout is summarized in Table 2.

### 2nd readout: CLNR detection without coronal STIR

Thirteen patients were correctly identified as having CLNR (true positives; TP), 710 as not having CLNR (true negatives; TN). Three patients were falsely detected as having CLNR (false positives; FP) and 25 as not having CLNR (false negatives; FN). This translates into a sensitivity of 34.2%, specificity of 99.6%, positive predictive value (PPV) of 81.3%, negative predictive value (NPV) of 96.6%, and accuracy of 96.3% without coronal STIR imaging. All TP and FP patients were initially detected with the axial T2-weighted sequence; none of them was primarily detected with the sagittal acquisitions. Six of the TP were additionally seen with a second look on the sagittal T1-weighted images, whereas none of the FP was detected on the sagittal images with a second look. Regarding the 25 FN patients, in the original consensus reading, 19/25 (76%) were detectable on the axial T2-weighted, 7/25 (28%) on the sagittal T1-weighted, and 25/25 (100%) on the coronal STIR images. Of these 25 missed CLNR patients, only 5/25 (20%) could simultaneously be detected on the sagittal and axial acquisitions in the consensus reading. Regarding the 5 patients with bilateral CLNR, only the ones with CLNR affecting multiple segments (2/5; 40%) were successfully detected in this readout (Fig. 3 and 4). Inter-reader agreement for CLNR detection without the coronal STIR was moderate (*κ* = 0.592; 95% CI 0.38, 0.80). Overall detection rate of CLNR and detection rate according to subtype in comparison to the 3rd readout are summarized in Table 3.

**Table 3:** **CLNR detection rate in the 2nd (no coronal STIR) vs. 3rd (with coronal STIR) readout**

CLNR* in consensus(*n*)	2nd readout *n* (%)	3rd readout *n* (%)	*P*-value^1^
Ia (5)	1 (20)	5 (100)	.13
Ib (21)	12 (57.1)	19/21 (90.5)	.016
IIa (0)	0 (0)	0 (0)	N/A
IIb (14)	1 (7.1)	9 (64.3)	.008
III (3)	1 (33.3)	2 (66.6)	1.0
All CLNR (43)	15 (34.9)	35 (81.4)	** <.**001
All patients (38)	13 (34.2)	31 (81.6)	** <.**001

*CLNR*, conjoined lumbosacral nerve root;* N/A*, not applicable

^*1*^*Significant results (P* < *.05) are bolded*

^*^Classified according to Neidre and McNab[18]

### 3rd readout: CLNR detection with coronal STIR

Thirty-one patients were correctly identified as having CLNR (TP) and 712 patients as not having CLNR (TN). One patient was falsely detected as having CLNR (FP) and 7 patients as not having CLNR (FN). This translates into a sensitivity of 81.6%, specificity of 99.9%, positive predictive value (PPV) of 96.9%, negative predictive value (NPV) of 99.0%, and an overall accuracy of 98.9% for CLNR detection in lumbar spine MRI with a coronal STIR sequence. All TP patients were initially detected on the coronal STIR (19/31, 61.3%) or the axial T2-weighted sequence (12/31, 38.7%); none of them was primarily detected with the sagittal acquisitions. Thirteen of the TP (13/31, 41.9%) were additionally seen with a second look on the sagittal T1-weighted images, whereas none of the FPs was detected on the sagittal images with a second look. Regarding the 7 FN patients, in the original consensus reading, 4/7 (57.1%) were detectable on the axial T2-weighted, 0/7 (0%) on the sagittal T1-weighted, and 7/7 (100%) on the coronal STIR images. Of these 7 missed CLNR patients, 4/7 (57.1%) could simultaneously be detected on the sagittal and axial acquisitions in the consensus reading. Regarding the 5 patients with bilateral CLNR, all the ones with multiple affected segments and 2 of the 3 with the same segment affected bilaterally (4/5; 80%) were successfully detected in this readout (Figs. 3 and 4). Inter-reader agreement for CLNR detection with the coronal STIR was almost perfect (*κ* = 0.915; 95% CI 0.84, 0.99). Overall detection rate of CLNR and detection rate according to subtype in comparison to the 2nd readout are summarized in Table 3.

LSTV had a prevalence of 14.0% (105 patients, 54 females) in the study cohort, with no significant gender predilection (*p* = 0.15). Among affected patients, the most common subtypes according to Castellvi in descending frequency were IIa, IIIb, and IIb, accounting for almost 75% (Table 4**, **Fig. 7). About two-thirds of all LSTV (67/105, 63.8%) were L5 sacralizations. S1 lumbarization was less common (38/105, 36.2%). LSTV prevalence was 13.3% among patients without CLNR and 26.3% among patients with CLNR, revealing a weak (φ_c_ = 0.1) yet significant (*p* = 0.025) correlation between MRI-detected LSTV and CLNR. An almost perfect inter-reader agreement was found for the assessment of the LSTV (*κ* = 0.93; 95% CI 0.88, 0.96).

**Table 4:** **Subtype frequency of lumbosacral transitional vertebrae (LSTV)**

LSTV subtype*	(*n*, %)
Ia	4 (3.8)
Ib	10 (9.5)
IIa	41 (39.0)
IIb	12 (11.4)
IIIa	7 (6.7)
IIIb	25 (23.8)
IV	6 (5.7)

*LSTV subtypes were classified according to Castellvi [19]: I—dysplastic transverse process of the LSTV without lumbosacral pseudoarticulation; II—lumbosacral pseudarticulation of the LSTV transverse process; III—lumbosacral fusion of the LSTV transverse process; IV—LSTV bilaterally combining subtypes II and III. The letter “a” denotes unilaterally, and the letter “b” denotes bilaterally affected transverse processes

## Discussion

With 751 included patients, ours is the largest single-center study that has assessed the detection of CLNR on MR imaging to yet. The lumbar segment L5/S1 was affected most frequently (26/43, 60.5%) by CLNR, with no predilection for gender or side, in agreement with previous reports [1, 2]. A CLNR prevalence of 5.1% (38/751) was found. This value is within the reported range of prevalence values, ranging from 1.3%, in surgical, to 14%, in cadaver series [4, 5]. Interestingly, our reported frequency of CLNR on MRI is higher than for CT (2%) [9, 10] and myelography (2–4%) [5]. The discrepancy between imaging, surgical, and cadaver studies suggests an underestimation of CLNR in vivo [4]. Our MR imaging study with coronal STIR thus far is reaching the closest in vivo approximation in a large cohort, when compared to the cadaver studies.

Based on a small MRI case series by Gomez et al. (*n* = 5) and Böttcher et al. (*n* = 5), experts recommend MRI-assessment of CLNR with T2-weighted coronal sequence inclusion [2]. Our study results are consistent with these suggestions, with a significant increase in sensitivity (34.2 to 81.6%) and PPV (81.3 to 96.9%) when the coronal STIR is added to the routine MRI protocol, compared to sagittal and axial acquisitions alone. This is especially noteworthy, because specificity (99.6 to 99.9%), NPV (96.6 to 99.0%) and diagnostic accuracy (96.3 to 98.9%) are all slightly improved as well. With the added coronal STIR, inter-reader agreement improved from moderate to almost perfect, further showing that coronal STIR is a key sequence for CLNR diagnosis on MRI.

Consistent with the consensus reading, where coronal STIR was evaluated as most helpful for initial CLNR detection in 62.7% (27/43), detection of all CLNR subtypes in the study profited from coronal STIR imaging (*p* < 0.001) in the 3rd readout. In raw numbers, CLNR type Ib and IIb profited most strongly, as they were overall the most prevalent (35/43, 81.4%). In terms of percentage, type Ia (5/43, 11.6%) profited the most with an increase in detection rate by 80%. At first glance, this is somewhat surprising, as the 5 cases were all detectable on three imaging planes in the consensus, except for one case not being visible on the sagittal acquisition. However, in 4 cases the coronal and in 1 case the axial plane were considered most helpful for initial CLNR detection, with the remaining sequences, thus playing supporting roles in secondary validation and helping to avoid false positives.

The sagittal plane had the lowest CLNR detection rate in consensus (16/43, 37.2%), not once considered the most helpful for initial CLNR detection. Not a single CLNR was exclusively detectable on sagittal imaging. This contrasts with the findings by Kang et al. (*n* = 11), describing the sagittal shoulder sign with a mean frequency of 90.9%, surpassing their axial findings with a mean frequency of 59.1% [8]. These discrepancies can be explained by the small study cohort of the latter study, impairing generalizability. Furthermore, their sole axial discriminator for CLNR diagnosis was the passage of two nerve roots through the intervertebral foramen [8]. In contrast, our study employed several criteria on axial T2 TSE, allowing an axial CLNR detectability in consensus of 83.7% (36/43).

According to the scarce literature on the topic, bilateral CLNR are thought to be rare [21], which is in agreement with our findings, where only 13.2% of the CLNR patients had bilateral CLNR. CLNR diagnosis mostly relies on asymmetry, so especially bilateral same-segment CLNR cases are hard to detect, potentially causing them to be even more under-diagnosed, which is suggested by considerable variance in their reported prevalence [21–23]. Adding the coronal STIR sequence, their detection rate was doubled from the 2nd to 3rd readout, by improving symmetry assessment and better depiction of the nerve root course.

LSTV prevalence in our cohort of 14.0% (105/751) is in the middle of the range reported in large cohort studies by Byvaltsev et al. (*n* = 4816) and Ucar et al. (*n* = 3607), with a 8.1 to 18.9% prevalence, respectively [24, 25]. Almost perfect inter-reader agreement for LSTV on coronal STIR and a clear predominance of L5 sacralization in our study (67/105, 63.8%) were consistent with the literature [25, 26]. An interesting new observation is that LSTV was twice as frequent among patients with CLNR compared to patients without CLNR (26.3 vs. 13.3%). Thus, with LSTV being more common than CLNR, it would be worth having a second look for CLNR when an LSTV is encountered by the radiologist.

Regarding technical aspects, the coronal STIR sequence accounted for a significant part of the acquisition time of 20% (1:54 min per patient) of the scan protocol. Aside from offering reproducible assessment of CLNR and LSTV in this study, coronal STIR of the lumbar spine has been previously shown to facilitate detection of clinically relevant co-findings related to lower back pain, such as Modic Typ I endplate changes, paravertebral edema, inflammatory sacroiliac joint changes, and lumbo-sacral insufficiency fractures [12–14].

Limitations of this study need to be addressed. Firstly, we relied on lumbar spine MRI-based consensus as a reference standard, instead of surgical correlation. However, surgical findings are not more reliable than imaging for CLNR detection [4]. Cadaveric assessment [5] is an option as a gold standard, but it was beyond the scope of this retrospective single-center study. Nevertheless, it is important to acknowledge that our reference standard was one of the techniques being compared. Secondly, our deployed coronal STIR standard slice thickness of 4 mm in lumbar spine MRI is at the upper limit of recommendations by national and international radiological societies [15, 16]. Small structures like CLNR and supernumerary rootlets can be missed due to partial volume effects. They might be more accurately detected by deploying slicing ≤ 3 mm, as recommended by the European Society of Musculoskeletal Radiology [27]. Furthermore, in our institutional protocol, the coronal sequence was always acquired with a STIR contrast. It would be interesting to see in future studies how well coronal T2-weighted non-fat–suppressed imaging compares to coronal STIR imaging in the CLNR detection. The exclusion of preoperated patients, e.g., post-spondylodesis, represents a selection bias, since arising metal artifacts [28] in proximity to the lumbar nerve roots might lower diagnostic accuracy. Furthermore, since whole-spine scout acquisition was not included in our MRI protocol, LSTV was classified under the assumption of 12 paired thoracic ribs. This might impair the accuracy of LSTV assessment. However, our results for both prevalence and subtype frequency aligned well with the literature [24, 25]. Finally, based on our study setup, the study does not give further insight into the clinical implications of the additionally detected CLNR. Based on our positive results regarding CLNR accuracy with coronal STIR imaging of the lumbar spine, this would be a promising topic for a comparative cohort follow-up study, including the coronal STIR sequence.

In conclusion, this study indicates a prevalence of CLNRs of 5.1% on lumbar spine MRI. Coronal STIR allowed a significant increase in sensitivity from 34.2 to 81.6% and improved reproducibility. Furthermore, the presence of lumbosacral transitional vertebra on lumbar spine MRI may warrant a second look for CLNR, as the entities are significantly correlated.

## Supplementary Information

Below is the link to the electronic supplementary material.

ESM 1 (DOCX 213 KB

ESM 2 (DOCX 16.5 KB)

## Data Availability

Upon request, the authors can send documentation or raw data in order to verify the validity of their results.

## Abbreviations

CLNR: Conjoined lumbosacral nerve root
FN: False negative
FP: False positive
LSTV: Lumbosacral transitional vertebra
NPV: Negative predictive value
PPV: Positive predictive value
ROI: Region of interest
STIR: Short tau inversion recovery
TN: True negative
TP: True positive
TSE: Turbo spin echo
T1w: T1-weighted
T2w: T2-weighted
