# Supplementary material for: Diagnostic value of a coronal STIR sequence in conjoined lumbar nerve root detection: an MRI accuracy study
Source: Skeletal Radiol. 2025 May 14;54(10):2157–68. doi: 10.1007/s00256-025-04945-y (PMC12361294; doi:10.1007/s00256-025-04945-y)
Supplement: Supplementary file 1 — (DOCX 213 KB [file 256_2025_4945_MOESM1_ESM.docx]

**Supplementary Figures:**

**
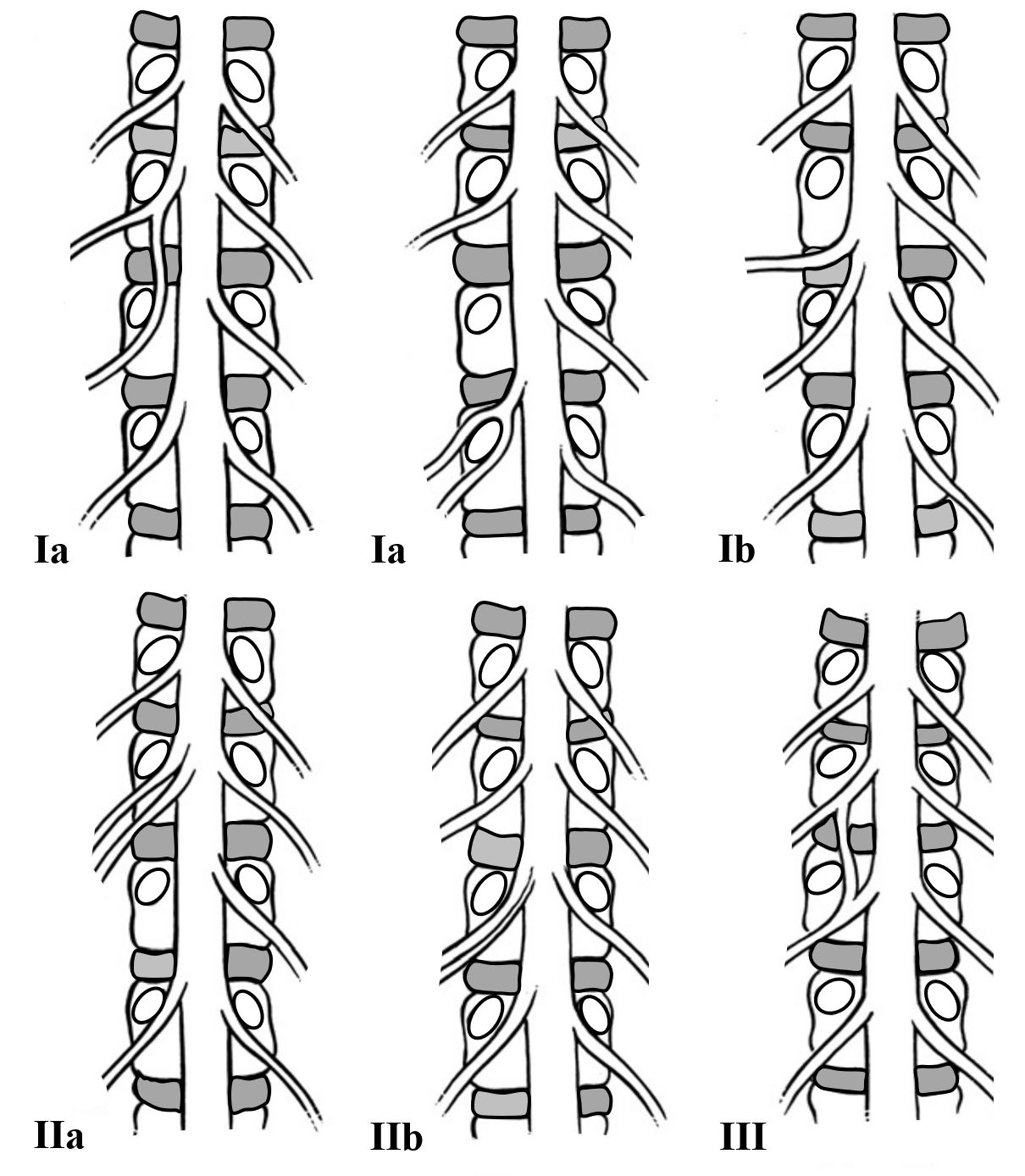
**

**Supplementary Fig. 1:** Illustration of conjoined lumbosacral nerve roots (CLNR) according to Neidre and McNab (14), dependent on their exit from the thecal sac.

**Type Ia**: Two nerve roots in a common dural sleeve with epidural division and both roots exiting separately through their respective foramina; two different variants are shown, the first with a more proximal division, and the second with a more distal division directly cranial of the pedicle. **Type Ib**: Two nerve roots in separate dural sleeves leaving the thecal sac in immediate proximity, exiting separately through their respective foramina (C). **Type** **IIa**: Two roots in seperate dural sleeves leaving the thecal in immediate proximity and exiting the through the same foramen, leaving an adjacent foramen above or below empty (D). **Type IIb**: Two rootlets in separate dural sleeves leaving the thecal sac in immediate proximity and exiting together through the same foramen with no adjacent empty foramina (E). **Type III**: Anastomosis between two adjacent nerve roots.
