# Supplementary material for: Diagnostic value of a coronal STIR sequence in conjoined lumbar nerve root detection: an MRI accuracy study
Source: Skeletal Radiol. 2025 May 14;54(10):2157–68. doi: 10.1007/s00256-025-04945-y (PMC12361294; doi:10.1007/s00256-025-04945-y)
Supplement: Supplementary file 2 — (DOCX 16.5 KB) [file 256_2025_4945_MOESM2_ESM.docx]

**Supplementary Tables:**

**Supplementary Table 1:** Lumbar spine MRI indications in 751 patients.

| **MRI indication (n, %)** | **(n, %)** |
| --- | --- |
| Acute, sub-chronic or chronic lower back pain without radiculopathy | 414 (55.1) |
| Acute, sub-chronic or chronic lower back pain with suspected radiculopathy | 210 (28.0 |
| Neurogenic claudication/ pseudoclaudication | 57 (7.5) |
| Sciatica | 45 (6.0) |
| Acute trauma | 15 (2.0) |
| Cauda equina syndrome | 5 (0.7) |
| Polyneuropathy | 5 (0.7) |
